# Overexpression of Banana *ATG8f* Modulates Drought Stress Resistance in *Arabidopsis*

**DOI:** 10.3390/biom9120814

**Published:** 2019-12-02

**Authors:** Bing Li, Guoyin Liu, Yuqi Wang, Yunxie Wei, Haitao Shi

**Affiliations:** 1 Hainan Key Laboratory for Sustainable Utilization of Tropical Bioresources, College of Tropical Crops, Hainan University, Haikou 570228, China; 17090102210006@hainanu.edu.cn (B.L.); luxy1212@163.com (G.L.); 2 College of Forestry, Hainan University, Haikou 570228, China; wangyuqiya@163.com

**Keywords:** autophagy, *MaATG8f*, drought, abscisic acid, reactive oxygen species, banana

## Abstract

Autophagy is essential for plant growth, development, and stress resistance. However, the involvement of banana autophagy-related genes in drought stress response and the underlying mechanism remain elusive. In this study, we found that the transcripts of 10 banana *ATG8s* responded to drought stress in different ways, and *MaATG8f* with the highest transcript in response to drought stress among them was chosen for functional analysis. Overexpression of *MaATG8f* improved drought stress resistance in *Arabidopsis*, with lower malonaldehyde level and higher level of assimilation rate. On the one hand, overexpression of *MaATG8f* activated the activities of superoxide dismutase, catalase, and peroxidase under drought stress conditions, so as to regulate reactive oxygen species accumulation. On the other hand, *MaATG8f*-overexpressing lines exhibited higher endogenous abscisic acid (ABA) level and more sensitivity to abscisic acid. Notably, the autophagosomes as visualized by CaMV35S::GFP–MaATG8f was activated after ABA treatment. Taken together, overexpression of *MaATG8f* positively regulated plant drought stress resistance through modulating reactive oxygen species metabolism, abscisic acid biosynthesis, and autophagic activity.

## 1. Introduction

Drought stress is one of the most serious environmental threats, resulting in large yield loss in agriculture [1]. With the change of global warming, drought stress is becoming more and more serious through influencing the dynamic regulation between crops and soil [2]. In order to survive under adverse environmental stresses such as drought stress, plants have developed complex mechanisms [3].

So far, abscisic acid (ABA) is widely known for its involvement in plant drought stress responses. ABA was firstly found in wheat under drought stress conditions [4]. Thereafter, the correlation between ABA and drought stress has been studied and proved in sugarcane [5], sorghum [6], maize [6], rice [7], barley [8], soybean [9], sunflower [10], and cowpea [11]. Endogenous ABA is largely and quickly induced in leaves under drought stress in plants [11]. Many genes, such as *Arabidopsis ABA insensitive 1* (*AtABI1*), modulate drought stress resistance in the ABA-signaling pathway [12,13]. In addition, the increase of ABA level enhances plant tolerance to cold, waterlogging, and salt stresses [14]. In recent decades, more and more studies have shown the relationship between autophagy and ABA in plants, especially under drought stress conditions [15,16,17,18].

As a conserved procession, autophagy participates in the protein degradation pathway in lysosome and vacuole [19,20]. In this process, substances to be degraded such as misfolded proteins in the cell are surrounded by autophagosome possessing bilaminar membrane, and then digested by vacuole [21]. As ubiquitin-like proteins, ATG8 proteins are processed by ATG4 proteins and combine with phosphatidylethanolamine [22]. The assemblies of ATG8 proteins are anchored in the phagophore to recruit proteins for further expansion of phagophore [23]. ATG8 proteins are also involved in the fusion of autophagosomes with lysosomes and in the transport of autophagosomes [24]. Additionally, autophagy has been widely investigated in model plants and some important crops [25,26]. Autophagy is involved in plant growth and development, such as leaf senescence, seed development, reproductive development, and vascular development [27,28,29,30]. For example, deficiency of *atg5* and *atg7* increases the root meristem growth under high glucose stress conditions [31], and deficiency of *atg9* leads to premature senescence and the accumulation of autophagosome in endoplasmic reticulum [32,33]. Moreover, autophagy is involved in plant stress responses. On the one hand, overexpression of *Malus domestica MdATG3a*, *MdATG3b*, *MdATG8i* in *Arabidopsis* and overexpression of *MdATG18a* in apple plants increase plant resistance to osmotic stress, deficiency of nutrient, and drought, respectively [16,17,34]. On the other hand, deficiency of *Atatg18a*, *Zmatg12*, *Osatg10b*, and *Atatg2/Atatg5/Atatg7/Atatg10* decrease plant stress resistance to heat, nitrogen deficiency, and hypoxia, respectively [25,35,36,37]. Moreover, deficiency of *ATGs* causes stagnation of protein degradation, stress of endoplasmic reticulum, and cell death [38]. Reactive oxygen species (ROS), which are quickly and largely burst under stress conditions, are also closely related to autophagy [39].

The number of subfamilies of *ATG8s* varies in different species. Compared with one *ATG8* in yeast (*Saccharomyces cerevisiae*), there are 9 members in *Arabidopsis thaliana* [40], 6 members in rice (*Oryza sativa* L.) [26], 13 members in wheat (*Triticum aestivum* L.) [41], 3 members in barley (*Hordeum vulgare* L.) [42], 5 members in pepper (*Capsicum annuum* L.) [43], and 10 members in banana (*Musa acuminata*) [44]. At present, the involvement of *MaATGs* in drought stress response and the underlying mechanism remain unclear. As one of the most favored fruits in the world, banana is an essential economic crop in tropical or sub-tropical areas, and water loss is quicker in tropical or sub-tropical areas than other regions. Therefore, it is very important for banana to withstand drought stress [45]. The objective of this study was to explore the function of *MaATG8f* in response to drought stress, so as to reveal its potential and vital improvement in drought stress resistance.

## 2. Materials and Methods

### 2.1. Plant Transformation and Screening of Transgenic Plants

The recombinant plasmid pEGAD–*MaATG8f* (*PromoterCaMV35S::GFP*–*MaATG8f*) has been described previously [44,46]. Briefly, the sequence of *MaATG8f* was cloned into pEGAD vector in the C-terminal in frame with green fluorescent protein (GFP) to form the constructs of *PromoterCaMV35S::GFP*–*MaATG8s*. In this study, *PromoterCaMV35S::GFP*–*MaATG8f* was introduced into *Agrobacterium tumefaciens* strain GV3101 and transformed into the wild-type (WT, Col-0) *Arabidopsis*, according to the reported method [47]. The *MaATG8f*-overexpressing lines were selected by Basta resistance and confirmed by PCR and quantitative real-time PCR (qRT-PCR) analysis. The primers used are listed in Table S1.

### 2.2. Plant Materials and Treatments

Banana seedlings and *Arabidopsis* seedlings (WT and *MaATG8f*-overexpressing lines) were cultivated in the mixed soil (vermiculite/nutrition soil = 2:1, *V*:*V*) in green house at 25 ± 1 °C, with 16 h light/8 h dark cycles. For drought stress treatment, about 1-month-old banana and *Arabidopsis* seedlings in the soil were withheld water for designed days, while the control seedlings were watered every 4 days. At designed time-points of treatments, banana or *Arabidopsis* leaves were collected for the assays of physiological parameters.

### 2.3. RNA Extraction and qRT-PCR

Total RNA extraction, purification, and first-strand cDNA synthesis were carried out by using the RNAprep Pure Plant Plus Kit (TIANGEN, DP441, Beijing, China), RNase-freeDNase (NEB, M0303S, Ipswich, MA, USA), and the Revert Aid First Strand cDNA Synthesis Kit (Thermo Scientific, K1622, Waltham, MA, USA), respectively. All procedures were conducted according to the manufacturer’s instructions. The mixture of cDNA, primers, and TransStart Tip Green qPCR SuperMix (TransGen Biotech, AQ141, Beijing, China) were reacted in the LightCycler ^®^ 96 Real-Time PCR System (Roche, Basel, Switzerland) for quantitative real-time PCR. All the transcript levels were analyzed using the comparative ΔΔCt method in comparison to the reference gene *Musa acuminata Ribosomal Protein S* (*MaRPS*) [48]. The primers used are listed in Table S1.

### 2.4. Determination of ROS and Malondialdehyde (MDA), and Activities of Antioxidant Enzymes

Herein, 1 mg/mL Di-amino benzidine (DAB) and 1 mg/mL nitroblue tetrazolium (NBT) were used to stain the 5th to 8th rosette leaves from 7-week-old seedlings for H_2_O_2_ and O_2_^−^, respectively [49], according to the previous method [16]. The measurements of H_2_O_2_, O_2_^−^, and MDA were performed according to the previous studies [50,51]. Briefly, leaf samples used for determination of H_2_O_2_ and O_2_^−^ were extracted by 50 mmol/L phosphate buffer. Then, the supernatant was mixed with titanium sulphate and determined at 415 nm to calculate the concentration H_2_O_2_, while the supernatant was mixed with sulphanilic acid and α-naphthylamine and detected at the absorbance of 530 nm to determine the concentration of O_2_^−^. MDA was determined by trichloroacetic acid and thiobarbituric acid, and the absorbance was determined at 532, 600, and 450 nm.

In addition, the activities of superoxide dismutase (SOD), catalase (CAT), and peroxidase (POD) were detected using the Total Superoxide Dismutase Assay Kit (Nanjing Jiancheng, A001-1, Nanjing, China), the Catalase Assay Kit (Nanjing Jiancheng, A007-1, Nanjing, China), and the Peroxidase Assay Kit (Nanjing Jiancheng, A084-3, Nanjing, China), respectively. 

### 2.5. Measurement of Assimilation Rate 

The assimilation rate in the 5th to 8th leaves were determined using the Portable Photosynthesis System (Hansatech, CIRAS-3, Norfolk, UK) with 1.75 cm^2^ area chamber. The internal light PAR was set at 1200 μmol m^−2^ s^−1^, and the carbon dioxide concentration was 380–400 ppm. Then, the assimilation rate was directly calculated by CIRAS-3.

### 2.6. Analysis of ABA Sensitivity

ABA level was detected by ABA ELISA Kit (Haling, HL97003, Shanghai, China). Five-day-old seedlings were transferred on MS with 0, 1, 5, and 10 μmol/L ABA for 8 days to measure primary root length. Simultaneously, surface-sterilized seeds from each *MaATG8f*-overexpressing line and the control were cultured on MS with or without 0, 0.5, 1, and 2 μmol/L ABA for 6 days to record the ratio of green cotyledons.

### 2.7. Observation of Autophagosome

Five-day-old seedlings of WT and *MaATG8f*-overexpressing lines growing on MS medium were transferred into MS medium with or without 20 μmol/L ABA for 1 day. Then, the fluorescence at 900 ms through GFP channel in elongation zone of roots was examined by fluorescence microscope (Leica, DM5000, Wetzlar, Germany).

### 2.8. Statistical Analysis

There were at least three biological replicates for every experiment. All data in this study were analyzed using ANOVA and Student’s t-test, and expressed as means ± standard deviation (SD). The asterisk (*) was used to indicate the difference at *p* < 0.05 in comparison to WT.

## 3. Results 

### 3.1. The Transcript Levels of MaATG8s in Response to Drought Stress

**Figure 1:**
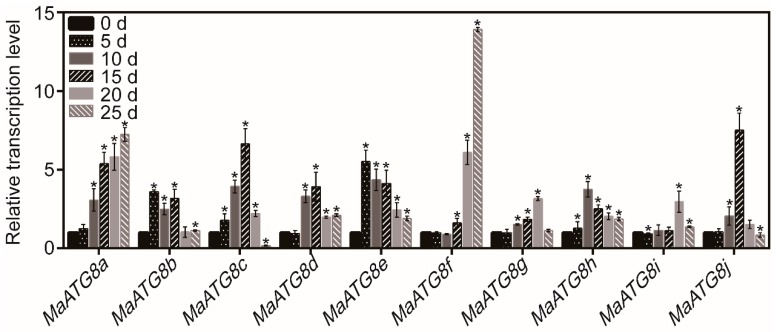
Analysis of the relative transcript levels of *MaATG8s* in response to drought stress. For the assay, 6-leaf-stage of banana, seedlings were withheld water for 0, 5, 10, 15, 20, and 25 days, respectively. *n* = 3, * *p* < 0.05.

First of all, the transcript levels of *MaATG8s* were analyzed by qRT-PCR under drought stress treatment for 0–25 days (Figure 1). Generally, the transcript levels of all *MaATGs* were regulated under drought stress conditions by different ways. Among them, the transcript level of *MaATG8f* showed a constant rising trend within 25 days, and it was much higher than the transcript levels of other *MaATG8s* at 25 days of drought stress treatment (Figure 1). Due to the highest expression in response to drought stress treatment, *MaATG8f* was chosen for functional analysis. 

### 3.2. Overexpression of MaATG8f Increases Drought Stress Resistance in Arabidopsis 

**Figure 2:**
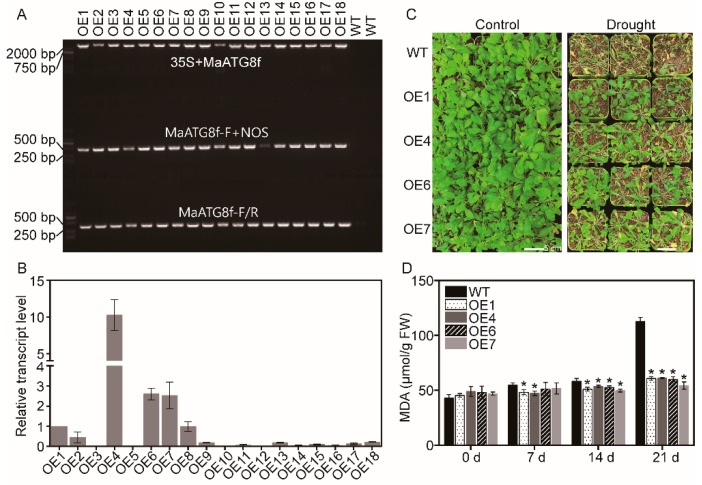
Phenotype of wild-type (WT) and *MaATG8f*-overexpressing *Arabidopsis* lines under drought stress conditions. (**A**) PCR verification in transgenic lines. (**B**) Relative transcript level of *MaATG8f* in *MaATG8f*-overexpressing lines. The transcript level of *MaATG8f* in OE1 was normalized as 1, which was used as control in qRT-PCR for other transgenic lines. (**C**) Phenotype of WT and overexpressing lines under drought stress conditions. Bars = 5 cm. (**D**) malondialdehyde (MDA) content in WT and *MaATG8f*-overexpressing leaves in response to drought stress. *n* = 3, * *p* < 0.05.

To investigate the function of *MaATG8f*, *MaATG8f* was overexpressed in *Arabidopsis*. In total, 18 *MaATG8f*-overexpressing lines were obtained and validated by PCR (Figure 2A), as well as qRT-PCR (Figure 2B). Based on the transcript levels of *MaATG8f* in the transgenic lines, four independent transgenic lines (OE1, OE4, OE6, and OE7) were selected for the analysis of phenotype. Under normal condition, there was no difference of phenotype among these four transgenic lines. Under drought stress conditions, the leaves of *MaATG8f*-overexpressing lines leaves were less shriveling and greener than that of WT (Figure 2C). Consistently, OE1, OE4, OE6, and OE7 showed significantly lower MDA level than WT during drought stress treatment (7, 14, and 21 days), especially at 21 days of drought stress treatment (Figure 2D). This result shows that *MaATG8f*-overexpressing lines exhibited much better performance than WT in response to drought stress, indicating the protective role of *MaATG8f* in drought stress response.

### 3.3. Determination of ROS and the Activity of Antioxidant Enzymes 

**Figure 3:**
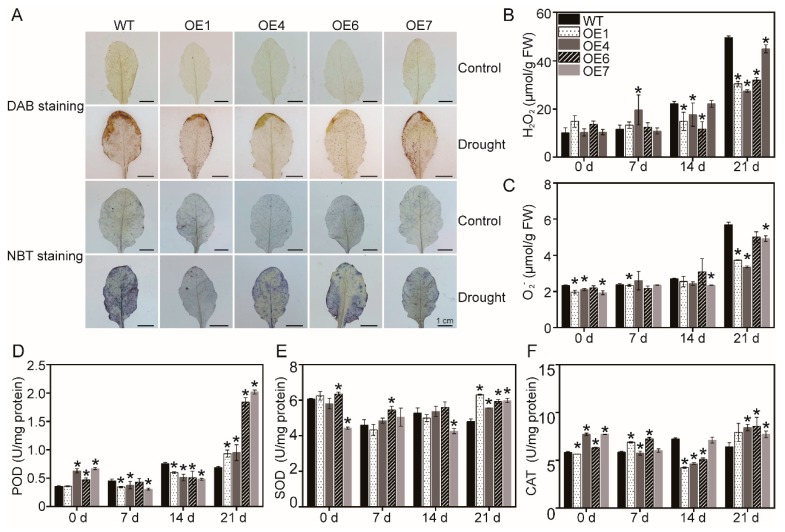
*MaATG8f* enhanced the resistance of overexpressing plants by regulating accumulation of reactive oxygen species and activities of antioxidant enzymes of WT and overexpressing lines during the 21-day drought treatment. (**A**) Di-amino benzidine (DAB) and nitroblue tetrazolium (NBT) staining. (**B**) H_2_O_2_ content. (**C**) O_2_^−^ content. (**D**) Peroxidase (POD) activity. (**E**) Superoxide dismutase (SOD) activity. (**F**) Catalase (CAT) activity. In these assays, the 5th to 8th leaves in the rosette were taken for the study. *n* = 3, * *p* < 0.05.

The endogenous H_2_O_2_ and O_2_^−^ levels that were visualized by DAB staining and NBT staining, respectively, can directly reflect the degree of oxidative damage in plants. Under control conditions, there were no significant differences in both brown color by DAB staining and blue zone by NBT staining between WT and *MaATG8f*-overexpressing leaves. Under drought stress conditions, *MaATG8f*-overexpressing leaves displayed lighter brown color by DAB staining and less blue zone by NBT staining than WT leaves (Figure 3A). Consistently, quantification of H_2_O_2_ (Figure 3B) and O_2_^−^ (Figure 3C) demonstrated that *MaATG8f*-overexpressing lines accumulated much lower levels of H_2_O_2_ and O_2_^−^ than WT in the leaves during drought stress. 

To further reveal the role of *MaATG8f* on the activity of antioxidant enzymes, three key enzymes (POD, SOD, and CAT) were chosen for analysis. SOD serves as the key antioxidant enzyme to catalyze O_2_^−^ into H_2_O_2_ and O_2_^−^, while POD and CAT can directly break down H_2_O_2_ to H_2_O [52]. Transgenic lines displayed significantly higher POD and SOD activities than WT upon drought stress (Figure 3D,E). In addition, CAT activity in *MaATG8f*-overexpressing lines was higher than that in WT (Figure 3F). Therefore, higher activity of these antioxidant enzymes might contribute to lower ROS accumulation in *MaATG8f*-overexpressing leaves in response to drought stress.

### 3.4. Overexpression of MaATG8f Promotes Photosynthesis Efficiency

**Figure 4:**
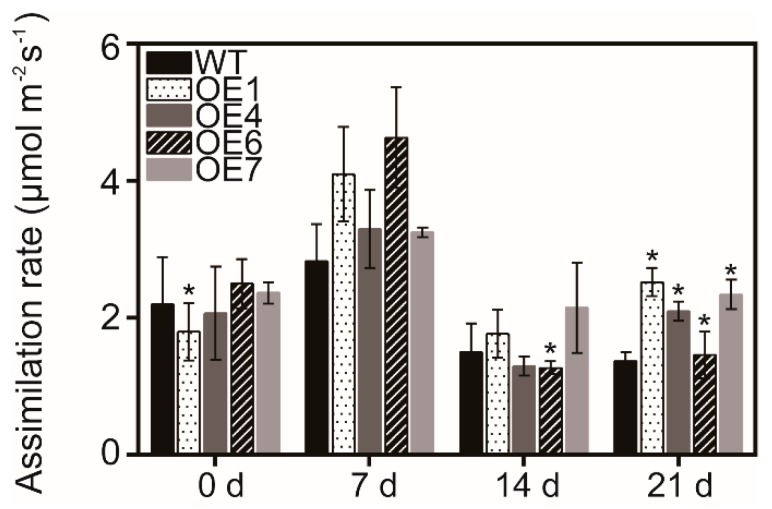
Assimilation rate in WT and *MaATG8f*-overexpressing leaves in response to drought stress. This assay was performed on sunny days between 10:00 and 12:00 h. Data are the means of five replicates with standard deviation (SD). *n* = 3, * *p* < 0.05.

To explore the relationship between autophagy and photosynthesis, assimilation rate was detected during drought stress treatment. Under drought stress conditions for 21 days, the photosynthesis rate in four overexpressing lines was significantly higher than WT leaves (Figure 4). This result indicates that overexpression of *MaATG8f* could maintain effective photosynthesis rate in response to drought stress.

### 3.5. Overexpression of MaATG8f Increases the Sensitivity to ABA in Arabidopsis

**Figure 5:**
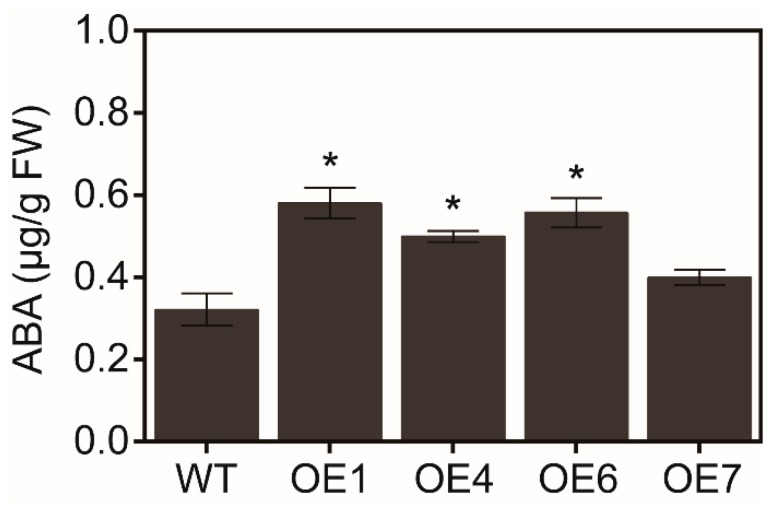
Endogenous abscisic acid (ABA) level in the leaves of 4-week-old WT and *MaATG8f*-overexpressing lines. *n* = 3, * *p* < 0.05.

**Figure 6:**
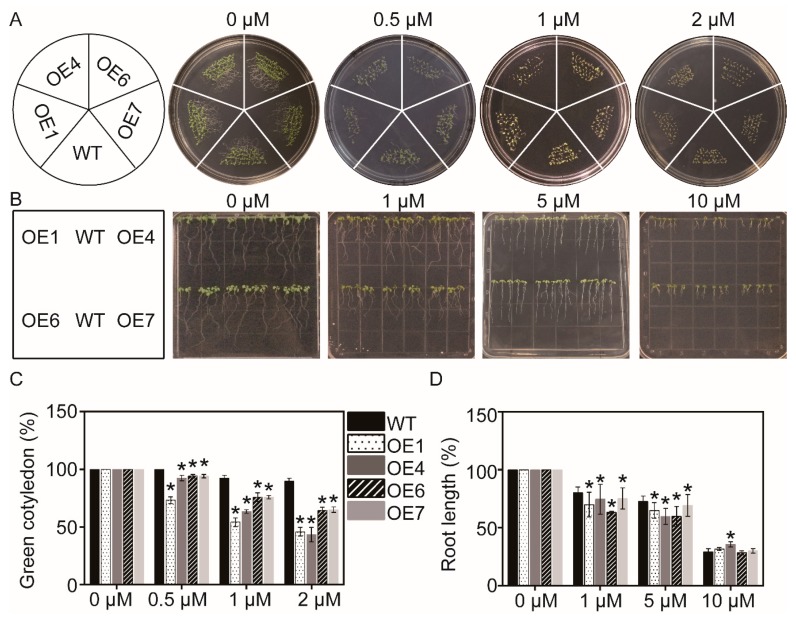
Assay of ABA sensitivity. (**A**) Phenotype of WT and *MaATG8f*-overexpressing lines in MS plate with 0, 0.5, 1, and 2 μmol/L ABA. (**B**) Root development of 4-day-old WT and overexpressing lines in MS plate with 0, 1, 5, and 10 μmol/L ABA. (**C**) Statistic of green cotyledon on day 6. (**D**) Relative primary root length in MS plate with different concentration of ABA for 6 days. *n* = 3, * *p* < 0.05.

Under control condition, the endogenous ABA level in *MaATG8f*-overexpressing leaves was significantly higher than that in WT leaves (Figure 5). When they grew on MS plate with different concentrations of ABA, *MaATG8f*-overexpressing lines were more sensitive to ABA, with lower green cotyledon rate and shorter primary root length (Figure 6A–D). Generally, *MaATG8f*-overexpressing lines regulated both endogenous ABA level and plant sensitivity to exogenous ABA.

### 3.6. Overexpression of MaATG8f Promotes the Generation of Autophagosomes byABA

**Figure 7:**
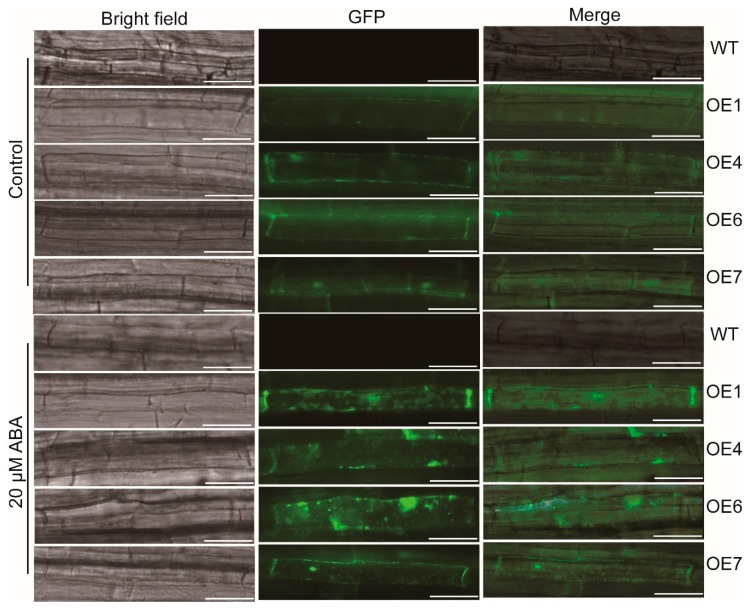
Observation of autophagosome under fluorescence microscope. For the assay, 7-day-old seedlings of WT and overexpressing lines (*CaMV35S::GFP-MaATG8f*) were transferred to MS plate with 0 and 20 μmol/L ABA for 1 day, and then the seedlings were transferred to the slide with 50 mM phosphate-buffered saline for analysis. The elongation zone of roots was observed under microscope (Leica, DM5000, Wetzlar, Germany) at 900 ms exposure intensity through GFP channel. Bars = 50 μm.

Under normal condition, the signal of GFP-MaATG8f was detected in the membrane and nucleus. When ABA was applied, the fluorescence of GFP-MaATG8f was visualized in both the membrane and cell cytoplasm. Notably, many fluorescent spots were induced by ABA in the elongation zone of *MaATG8f*-overexpressing roots (*CaMV35S::GFP-MaATG8f*) (Figure 7), indicating the generation of autophagosomes by ABA treatment. 

## 4. Discussion

Autophagy that is mediated by *ATGs* is vital for plant metabolism in plant stress resistance and defense responses [53]. To date, plant *ATGs* are widely involved in plant stress responses. High salt and osmotic stresses induce the transcript level of *AtATG18a*, and deficiency of *ATG18a* decreases the resistance to salt and drought [25]. Moreover, the transcript levels of nine *ATGs* in *Capsicum annuum* L. are upregulated under salt stress, and silence of *CaATG8c* decreases plant resistance to osmotic and salt stress [43]. On the contrary, overexpression of *ATGs* can enhance stress resistance in multiple plant species [16]. In our previous study, 32 *ATGs* were identified in banana. Among them, *MaATG8s* with 10 members is the largest subfamily and the transcripts of them were commonly regulated by *Fusarium oxysporum* f. sp. *cubense* [44]. Herein, the transcript level of most *MaATG8s* was upregulated by drought stress. Consistent with *ATGs* in other plant species, overexpression of *MaATG8f* in *Arabidopsis* enhanced the adaptation to drought stress and alleviated the damage of lipid peroxidation in plants, confirming that *MaATG8f* is a positive regulator of drought stress resistance. Therefore, the function of plant *ATG8s* may be conserved in multiple plants, and they may be used as ideal genes for stress related molecular breeding. 

ROS, including H_2_O_2_, O_2_^−^, and free radicals, can oxidize lipids, nucleic acids, and amino acid precursors, which could be considered as oxidative stress [54,55]. Oxidative stress leads to mitochondrial dysfunction and cell injury [56]. In this study, overexpression of *MaATG8f* alleviated the oxidative damage through modulation of ROS accumulation under drought stress conditions. The relationship between autophagy and ROS has been revealed. On the one hand, autophagy might be involved in degradation of ROS [31]. On the other hand, ROS could also initiate autophagosome formation and autophagic degradation, acting as cellular signaling molecules [57]. Meanwhile, autophagy can assist to scavenge oxidative damage and decrease ROS level through promoting the degradation of misfolded proteins and injured organelles [58]. Briefly, ROS plays an important role in the process of autophagy and is modulated by autophagy. Therefore, the dual roles of *MaATG8f* in scavenging the burst of ROS under drought stress conditions and triggering autophasomes might be directly related, and the complex effect resulted in improved drought stress resistance. 

Photosynthesis is restrained by drought stress in plants [59,60]. Herein, overexpression of *MaATG8f* in plants could keep photosynthesis with higher assimilation rate under drought stress conditions, which might contribute to improved drought stress resistance. This result is consistent with the higher assimilation rate in *MdATG18a*-overexpressing plants [16]. Therefore, overexpression of *ATG* keeps the efficiency of photosynthetic carbon assimilation, which may facilitate nutrient accumulation and promote carbon transportation under stress conditions. Chloroplasts are vital for solar light utilization and photosynthesis. Autophagy has a critical role in chloroplast degradation and might constitute a mechanism of dynamic control in chloroplasts [61]. Based on the vital role of autophagy in photosynthesis, the higher assimilation rate in *MaATG8f*-overexpressing lines might serve for better photosynthesis under drought stress conditions. In chloroplasts, ROS induced by abiotic stress break the balance of the redox system. In addition, the enhancement of assimilation in *MaATG8f*-overexpressing lines might also be a result of the improvement of the antioxidant system under drought stress. However, the underlying mechanism of autophagy in modulating the photosynthetic implementation or assisting in repairing important proteins needs to be investigated.

As an isoprenoid phytohormone, ABA regulates many plant stress responses, especially drought stress response [62]. Meanwhile, abiotic stress can activate ABA biosynthesis and the ABA signaling transduction pathway as a kind of systemic acquired acclimation [63]. Not only does ABA modulate stomatal closing to keep plant leaf water status and decrease water loss, but also the ABA signaling transduction pathway, including ABA receptor, the related kinases, transcription factors, and downstream genes, is involved in plant drought stress response [64,65,66,67,68,69,70]. Therefore, endogenous ABA level is directly related to plant leaf water status, and the activation of the key components in the ABA signaling transduction pathway is also related to plant drought stress resistance [71,72]. In this study, *MaATG8f*-overexpressing lines showed higher endogenous ABA level and more sensitivity to ABA than WT. Because of the essential role of ABA level and ABA sensitivity in plant drought stress response [64,65,66,67,68], the modulation of *MaATG8f* on ABA biosynthesis and ABA sensitivity might be directly related to its role in drought stress response. Although *MaATG8f* positively regulates drought stress resistance in the ABA-dependent pathway, the role of *MaATG8f* in the activation of the key components in the ABA signaling transduction pathway remains to be further investigated. 

Consistent with the induction of the transcripts of plant *ATGs* by multiple stresses and ABA [73], the formation of autophagosomes can also be significantly induced by them [37], but inhibited by the silencing of *ATGs* [74]. Autophagy can regulate the protein level of TSPO (tryptophan-rich sensory protein) in ABA signaling, especially under drought stress conditions [75]. Herein, we found that autophagosomes labeled by GFP-*MaATG8f* were activated by ABA treatment, indicating the involvement of *MaATG8f* and autophagy in ABA-mediated plant stress responses. We have to note that *MaATG8f* may have multiple targets, which may be regulated at the protein level. The identification of direct targets of *MaATG8f* may provide novel insight into the correlation between *MaATG8f*-mediated autophagy, ABA biosynthesis and signaling, as well as ROS metabolism. Although there may be some differences in the function of *MaATG8s* between *Arabidopsis* and banana, this study, together with other studies about plant *MaATG8s* [45], indicates that *MaATG8f* might be used as an ideal candidate for crop genetic breeding.

## 5. Conclusions

In conclusion, this study has shown that overexpression of *MaATG8f* results in improved drought stress resistance through activating the activities of antioxidant enzymes, regulating ROS accumulation, and activating endogenous ABA level and autophagic activity. 

## Supplementary Materials

The following are available online at https://www.mdpi.com/2218-273X/9/12/814/s1. Table S1: Primers used in this experiment.
