# Supplementary material for: Overexpression of Banana ATG8f Modulates Drought Stress Resistance in Arabidopsis
Source: Biomolecules. 2019 Dec 2;9(12):814. doi: 10.3390/biom9120814 (PMC6995610; doi:10.3390/biom9120814)
Supplement: Supplementary file 1 [file biomolecules-09-00814-s001.pdf]

Supplemental Table 1. Primers used in this experiment.

Primer	Sequence
MaATG8f-F	CCCCCCGGGATGGCGAAGAGTTCCTTCAA
MaATG8f-R	CGCGGATCCTTAAACAGATCCAAATGTGT
q*MaATG8a*-F	AACACCTTCGGAAGAGGATTAAG
q*MaATG8a*-R	TTCTTGACAAAAACGAAGATGG
q*MaATG8b*-F	TCAAGTTGGAACATCCTCTCG
q*MaATG8b*-R	CAGGATACTTCTCCCTGATACGA
q*MaATG8c*-F	TTCTGCTCTTTAGATGTAGCCTCA
q*MaATG8c*-R	CCAGAGTTGAAAGTAGCGAGATT
q*MaATG8d*-F	AACACGTTGCCACCAACAG
q*MaATG8d*-R	CCATCTTCATCTTTGTATTCTTCG
q*MaATG8e*-F	CCTCTTACGACGATATGCCTGT
q*MaATG8e*-R	ACCCAAGTTTACTAGGCAACAACT
q*MaATG8f*-F	CTGTTCATGCACCATTTCCA
q*MaATG8f*-R	GCGTCACAAGTTCGAACACA
q*MaATG8g*-F	AGGCCATCTTCGTCTTTGTG
q*MaATG8g*-R	TTCCTCGTAGATCGCAGACA
q*MaATG8h*-F	GCTACAAGCAGCAGCATGAC
q*MaATG8h*-R	TCTCCTTAATCCTCTGCGACTC
q*MaATG8i*-F	AAATACCCTGGCAGGTTTCC
q*MaATG8i*-R	TCAATATTTGGCACATCACTTGT
q*MaATG8j*-F	TCGCCATGAAGCAGAAGT
q*MaATG8j*-R	GTGAATAAATTGCCCAACA
